# Extracellular PHF-tau modulates astrocyte mitochondrial dynamics and mediates neuronal connectivity

**DOI:** 10.1186/s40035-025-00474-9

**Published:** 2025-03-24

**Authors:** Valentin Zufferey, Aatmika Barve, Enea Parietti, Luc Belinga, Audrey Bringaud, Yvan Varisco, Kerstin Fabbri, Francesca Capotosti, Paola Bezzi, Nicole Déglon, Pierre Marquet, Nicolas Preitner, Kevin Richetin

**Affiliations:** 1 Centre for Psychiatric Neurosciences (CNP), Lausanne University Hospital (CHUV) - University of Lausanne (UNIL), 1015 Lausanne, Switzerland; 2 Leenaards Memory Centre, Lausanne University Hospital (CHUV) - University of Lausanne (UNIL), 1011 Lausanne, Switzerland; 3 Department of Clinical Neuroscience (DNC), Laboratory of Neurotherapies and Neuromodulation, Lausanne University Hospital (CHUV) and University of Lausanne, 1011 Lausanne, Switzerland; 4 Service for Autism Spectrum Disorders (STSA), Department of Psychiatry, Lausanne University Hospital (CHUV), 1011 Lausanne, Switzerland; 5 AC Immune SA, 1015 Lausanne, Switzerland; 6 Department of Fundamental Neurosciences, University of Lausanne (UNIL), 1005 Lausanne, Switzerland

**Keywords:** Tau, Mitochondria, Astrocytes, Synapse, Live imaging microscopy

## Abstract

**Background:**

Tau is an intracellular protein that plays a crucial role in stabilizing microtubules. However, it can aggregate into various forms under pathological conditions and be secreted into the brain parenchyma. While the consequences of tau aggregation within neurons have been extensively studied, the effects of extracellular paired helical filaments of tau (ePHF-tau) on neurons and astrocytes are still poorly understood.

**Methods:**

This study examined the effect of human ePHF-tau (2N4R) on primary cultures of rat neuroglia, focusing on changes in neurites or synapses by microscopy and analysis of synaptosome and mitochondria proteomic profiles after treatment. In addition, we monitored the behavior of mitochondria in neurons and astrocytes separately over three days using high-speed imaging and high-throughput acquisition and analysis.

**Results:**

ePHF-tau was efficiently cleared by astrocytes within two days in a 3D neuron-astrocyte co-culture model. Treatment with ePHF-tau led to a rapid increase in synaptic vesicle production and active zones, suggesting a potential excitotoxic response. Proteomic analyses of synaptosomal and mitochondrial fractions revealed distinct mitochondrial stress adaptations: astrocytes exhibited elevated mitochondrial biogenesis and turnover, whereas neuronal mitochondria displayed only minor oxidative modifications. In a mixed culture model, overexpression of tau 1N4R specifically in astrocytes triggered a marked increase in mitochondrial biogenesis, coinciding with enhanced synaptic vesicle formation in dendrites. Similarly, astrocyte-specific overexpression of PGC1alpha produced a comparable pattern of synaptic vesicle production, indicating that astrocytic mitochondrial adaptation to ePHF-tau may significantly influence synaptic function.

**Conclusions:**

These findings suggest that the accumulation of PHF-tau within astrocytes drives changes in mitochondrial biogenesis, which may influence synaptic regulation. This astrocyte-mediated adaptation to tauopathy highlights the potential role of astrocytes in modulating synaptic dynamics in response to tau stress, opening avenues for therapeutic strategies aimed at astrocytic mechanisms in the context of neurodegenerative diseases.

**Supplementary Information:**

The online version contains supplementary material available at 10.1186/s40035-025-00474-9.

## Background

Under physiological conditions, the tau protein plays a crucial role in stabilizing microtubules within neurons, thereby preserving the cellular architecture and facilitating intracellular transport [1]. However, with aging and under the influence of pathological factors, tau can undergo post-translational modifications, notably abnormal hyperphosphorylation. This leads to its detachment from microtubules and aggregation into soluble and insoluble forms [2]. These aggregates include soluble oligomers, paired helical filaments (PHF-tau), and, ultimately, neurofibrillary tangles, composed of various species of phosphorylated Tau.

The formation of PHF-tau results from the self-aggregation of hyperphosphorylated tau into insoluble filaments that accumulate within neurons, disrupting microtubule dynamics and axonal transport [3]. Upon neuronal death, these intracellular aggregates can be released into the extracellular space, forming extracellular PHF-tau (ePHF-tau) [4]. ePHF-tau can adopt different conformations and levels of toxicity, influencing various pathological mechanisms in the brain [5, 6]. The different forms of hyperphosphorylated soluble tau [7], tau aggregates [8], and tau oligomers [9] have been described with varying degrees and mechanisms of toxicity. Soluble forms of tau, particularly oligomers, are considered especially neurotoxic, affecting synaptic plasticity, disrupting intracellular signaling, and inducing mitochondrial dysfunction [10–12]. These oligomers can interact with membrane receptors, disrupt calcium homeostasis, and induce oxidative stress [13, 14]

ePHF-tau can interact with surrounding cells, including neurons and glial cells, facilitating the trans-synaptic propagation of Tau pathology in the brain [15, 16]. This cell-to-cell transmission process has been extensively studied, revealing that ePHF-tau can be internalized by neighbouring cells through mechanisms such as receptor-mediated endocytosis or macropinocytosis, thereby altering the function of endogenous tau and compromising synaptic and mitochondrial functions [5, 17]. Furthermore, ePHF-tau can induce inflammatory responses, activate microglial and astrocytic cells, and contribute to synaptic dysfunction and cell death through mechanisms of excitotoxicity and oxidative stress [18, 19].

The tau protein, in its various pathological forms, can exert significant deleterious effects on mitochondria, thereby contributing to neuronal dysfunction in tauopathies such as Alzheimer's disease (AD) [20–22]. Specifically, the accumulation of hyperphosphorylated tau disrupts mitochondrial transport by increasing the frequency of mitochondrial pauses and reducing their anterograde movement, leading to insufficient energy supply at synapses and impairing synaptic transmission [23].

Additionally, truncated forms of tau, such as the Asp421 fragment cleaved by caspase-3, induce mitochondrial fragmentation, loss of mitochondrial membrane potential (MMP), and increased production of reactive oxygen species (ROS), exacerbating oxidative stress and metabolic dysfunction [11, 24]. These mitochondrial impairments also include bioenergetic failures, reduced calcium buffering capacity, and structural damage, resulting in maladaptive cellular responses, such as the excessive opening of the mitochondrial permeability transition pore, which promotes cell death [21].

Although most research has focused on the deleterious effects of intracellular tau aggregation on neurons, recent studies have shown that glial cells particularly astrocytes [25], can accumulate tau in its hyperphosphorylated and aggregated form in various contexts and pathologies [26–29]. Astrocytes can internalize extracellular tau, including ePHF-tau, which can impair their function, disrupt the mitochondrial system [19, 30], and contribute to the spread of tau pathology [31]. Additionally, this internalization can lead to inflammatory responses and exacerbate neuronal damage [19]. To deepen our understanding of the cellular and mitochondrial impacts of tau pathology, we explored the effect of ePHF-tau aggregates, primarily composed of non-phosphorylated 2N4R tau, on three-dimensional (3D) mixed cultures. We aimed to decipher the mechanisms by which ePHF-tau influences cellular function, using an integrative approach that combines synaptosome proteomics and real-time fluorescence imaging. Our study highlights the complexity and temporal dynamics of the effects of ePHF-Tau on different synaptic compartments, with a particular focus on the changes occurring in the mitochondrial system of astrocytes and their consequences on neuronal connectivity.

## Methods

### PHF-tau preparation and treatment

PHF-tau was prepared by isolating the sarkosyl-insoluble fraction from the frontal cortex of patients with AD as previously described [32]. Asterand Bioscience (now bioIVT, UK) and the CHUV Brain biobank (BB_063) provided the samples. 3D neuron-glia co-cultures were treated at 10 days in vitro (DIV10). PHF-tau dilutions were added to the cell medium and incubated for three days. In live imaging assays, the first round of image acquisition was carried out as a baseline measurement just before treatment and at the start of continuous monitoring. Cells treated with DMSO (Sigma, St. Louis, MO, D2438-10ML) served as a control. At 72 h, the cells were fixed with 4% paraformaldehyde (Sigma, 158127-500G) for 15 min at 37 °C and stored in PBS at 4 °C for further analysis.

### Lentiviral vector production and infection

Lentiviral vectors were concentrated by ultracentrifugation and resuspended in phosphate-buffered saline (dPBS, Gibco, Life Technologies, Zug, Switzerland) supplemented with 1% bovine serum albumin (BSA, Sigma-Aldrich, Buchs, Switzerland). The viral particle content in each batch was determined using a p24 antigen enzyme-linked immunosorbent assay (p24 ELISA, RETROtek; Kampenhout, Belgium). The stocks were stored at − 80 °C until use and diluted to 100,000 ng/mL in PBS/1% BSA. The dose used in this study was 15 ng p24 per 100,000 cells. The Lentiviral vectors used in this study have been previously described [29, 30, 33–35]. The gfaABC1D promoter was ligated to enhancer B(3) to generate the G1B3 promoter, which was subsequently cloned and inserted into the SIN-cPPT-gateway-WPRE-miR124T transfer plasmid. The plasmid contains four copies of the neuron-specific miRNA-124 target sequence (miR124T; full homology) to repress transgene expression in neurons [36], a woodchuck hepatitis virus B postregulatory element (WPRE) and a central polypurin tract (cPPT) to increase transgene expression, and a 400-nucleotide deletion in the long 3′ terminal repeat (self-inactivating vector) to increase biosafety. For neurons, the reporter gene contained a WPRE and the mouse PGK promoter. Co-cultures were infected at DIV5 with lentiviral vectors (LV-PGK-MitoTimer) and/or DIV8 with lentiviral vectors (LV-G1B3-MitoTimer, LV-G1B3-1N4R-V5, LV-G1B3- PGC-1α).

### Primary cell cultures

The hippocampus of Wistar rat embryos at embryonic day 17 (E17) was dissected, and the cells were dissociated with a neuronal dissociation kit (Miltenyi Biotec, Bergisch Gladbach, Germany; 130–092–628). The mixed cells were plated at a density of 350,000 cells/cm^2^ in 24-well glass-bottom plates (Ibidi, Gräfelfing, Germany; 82426) coated with Matrigel (Corning, Corning, NY, 356234) in 200 µmol/L DMEM (Gibco, Waltham, MA; 41965–039, with 25 mmol/L glucose) supplemented with 0.25% *L*-glutamine (Gibco, 25030081), 1% penicillin/streptomycin (Thermo Fisher Scientific, Waltham, MA; 15140–122) and 2% B27 (Gibco, 17504044) and incubated at 37 °C with 5% CO_2_.

### Immunofluorescence of cell cultures

All the incubations were carried out in PBST (Gibco, 10010–015) with 0.3% Triton X-100 (Sigma–Aldrich, X100-100ML) and 3% horse serum (Gibco, 16050–122). Primary antibody incubation was performed overnight with rabbit anti-VGLUT1 (Cell Signaling Technology, Danvers, MA; 1230.31; 1:500) and anti-PGC-1α (Abcam, Cambridge, United Kingdom; 54481-R166495-3, 1:500), goat anti-PSD95 (Abcam, ab12093; 1:500) and anti-V5 (Invitrogen, Carlsbad, CA; 460705, 1:500), mouse anti-Neuronal nuclei (Sigma, MAB337, 1:500), anti-glial fibrillary acidic protein (GFAP) (Dako, Nowy Sącz, Pologne; GA52461-2, 1:500) and anti-neurofilament light chain (NFL) (Invitrogen, 13–0400, 1:500). Secondary antibody incubation was conducted for 60 min at room temperature with Alexa Fluor 488-, 555- or 647-conjugated highly cross-adsorbed donkey anti-goat, donkey anti-rabbit or donkey anti-chicken antibodies (Invitrogen, A31573, A11055; Jackson IR, West Grove, PA; 703–545–155; 1:500). After additional incubation in 4',6-diamidino-2-phenylindole (DAPI) (Merck, Darmstadt, Allemagne; 268298; 1:5000), the cells were stored at 4 °C in PBS.

### Microscopy and image analysis

Live monitoring experiments were carried out in the proximity of neurospheroids with 40 × magnification on a Nikon Eclipse TI-2 Microscope to acquire images before treatment (baseline) and after ePHF-tau treatment for 3 days, every 2 h for Mitotimer (see Supplementary Methods) and every 30 min for the synaptic dye (BioTracker 510 green C2, Sigma-Aldrich, SCT132). Brightfield images for ePHF-tau clearance monitoring were taken every 4 min to obtain a high temporal resolution during the observation. Neurospheroid image acquisition was conducted using a Nikon-based (Ni-E) microscope equipped with spinning-disk confocal technology (CrestOptic, X-Light V3). We scanned all neurospheroids and their close surroundings (*n* = 25) in stacks (z-step 0.6 µm, 60 × magnification). Other immunostaining acquisitions were conducted as described for mitochondrial monitoring (Ti2 microscope, mosaic, 40 × , same X/Y locations). Image analyses were performed using the General analysis 3 (GA3) module, and artificial intelligence modules of Nikon’s NIS Elements. ePHF monitoring and clearance analysis was performed on brightfield images using segment.ai modules of NIS-model to segment and count ePHF aggregates. Analysis of synaptic dye was performed in highly connected surroundings of neurospheroids and consisted in the detection of bright spots (contrast-based). Spot detection was carried out to quantify the number of synaptic vesicles and their mean fluorescence in neurite projections. Cell peri-somatic regions and background area were subtracted from the region of interest (ROI) areas for calculation of density. Mitochondrial analysis was conducted as previously described [29, 30] (see Supplementary Methods), within small ROIs placed on distal cell processes to ensure selection of cellular endpoints. A minimum of 12 cellular processes were monitored for each image sequence. The measurements were further processed through our Mitotimer analysis pipeline, briefly, normalization to baseline and normalization to controls at each time points to compare conditions or cell types (Fig. 4c). The cultures were fixed immediately after acquisition. After an AI-based denoising operation, we selected ROIs on the cell projections formed by PSD95 on the edge of the neurospheroids. Active zones were estimated from the colocalization of VGLUT1 and PSD95 (shell volume). We measured the volumes of analyzed ROIs, individual and integrated VGLUT1 and PSD95, and active zones; the surface of PSD95; and the numbers of individual VGLUT1 and active zone objects. Immunofluorescent assays of cultured cells (stained with DAPI, NeuN, and GFAP to calculate surface signal coverage and normalized to the cell count) were carried out to assess homogeneous culture density and cell type abundances. The expression of tau-1N4R in infected astrocytes was validated by measuring the fluorescence intensities of anti-V5 staining in the peri-nuclear zone of astrocytes (GFAP^+^ nuclei). Mitochondrial transcription factor A (TFAM) mean intensity was analyzed in the nucleus of astrocytes (GFAP^+^) and grouped according to the peri-nuclear positivity for V5 or PGC-1α staining.

### Synaptosome and mitochondrial fraction extraction

The cells were rinsed several times, collected, and snap frozen. Homogenization was performed on ice in PBS. The samples were centrifuged at 1300 × *g* for 3 min at 4 °C to pull down the membrane fragments, nuclei, and cells. The supernatant was coupled with Anti-tomm22 micro-Beats (30 min at 4 °C) to isolate free mitochondria through magnetic-assisted cell sorting (MACS) (Miltenyi Biotec, Bergisch Gladbach, Germany; 130–096–946, 130–042–401). The mitochondria-depleted flow-through was centrifuged at 13,000 × *g* for 10 min at 4 °C to pellet synaptosomes (100 μL of PBS resuspension, stored frozen until further analysis).

### Proteomic data analysis

For preprocessing of proteomics data (see Supplementary Methods), a cutoff was used for the presence of a protein in each sample, which includes unique + Razor Peptide Score > 2 and an MS/MS Count > 2. Around 29% of the proteins were discarded in this process. We were provided with two kinds of quantitative values: (1) intensity-based absolute quantification (IBAQ) values, which are the sum of the intensities of all unique peptides for a protein divided by the number of theoretical tryptic peptides between six and 30 amino acids in length [37, 38], and (2) label-free quantification (LFQ) values, calculated according to the number of unique peptides of a protein on the total number of peptides. Additionally, we calculated the relative protein abundance as the sum of relative IBAQ (rIBAQ) of all proteins that were independently present in each replicate. We used Database for Annotation, Visualization and Integrated Discovery (DAVID) [39, 40] to extract functional annotations specific for the updated protein list for *Rattus norvegicus* from Rat Genome Database (RGD) [41]. Specifically, DAVID pulled annotations from Gene Ontology (GO) [42, 43] for biological process (BP), cellular components (CC), and molecular function (MF); Kyoto Encyclopedia of Genes and Genomes [44] for biological pathways, and Reactome [45] for specific biochemical reactions. Perseus (v2.1.2.0) [46] has been the fundamental tool for our subsequent qualitative and quantitative proteomic analysis. Our annotated proteomic data generated by Perseus were further processed for 2D-enrichment [47] to extract the relevant annotation terms which are found to be significantly changed because of the tau condition. We represented this enrichment analysis using a scatter plot and enriched terms were shown in red region, and depleted terms were shown in blue region. To evaluate the differential expression of proteins in effect of tau in our proteomic dataset, we used LFQ values to calculate the fold change between tau and DMSO. We used volcano plot for the representation of differentially expressed proteins in synaptosomal-enriched fraction (SEF) and mitochondrial-enriched fraction (MEF), in which enriched and depleted proteins in ePHF-tau condition were indicated in red and blue, respectively. We used rIBAQ values to represent the quantitative changes in individual proteins to evaluate the effect of tau. This comparison was represented using boxplots. Cytoscape (v_3.8.0) and NDEx (The Network Data Exchange) were used to generate the protein–protein interaction network [48–51]. Interactions among the modified proteins and their influence on tau regulation were extracted using the Signor Complete Rat network database [52] and the BioGRID Protein–Protein Interactions for *Rattus norvegicus* [53].

### Data visualization

Python (v3.12) was used to plot 2D enrichment graphs and Volcano plots for proteomics data. Python libraries used included: pandas [54], matplotlib [55], seaborn [56], adjustText [57], brokenaxes [58], and openpyxl [59]. GraphPad Prism (v-9.0) was used to create the line plots, bar plots and boxplots for our data, with asterisks (*) showing significant *P*-values. Cytoscape was used to create the interactome for the modified proteins. We used styling and database features to extract the information and visualize it.

### Statistical analysis

Values are presented as the mean ± SEM. Statistical analyses were performed on raw data with GraphPad Prism software. Shapiro–Wilk tests were performed to test distribution normality. The level of significance was set to *P* < 0.05. For synaptic active zone analysis, comparison of counts, surfaces, and volumes was performed with *t*-tests or Mann–Whitney for normally and non-normally distributed data, respectively. For immunohistochemical analysis, one-way ANOVA was used for optical density and mitochondrial data analysis. For mass spectrometry data analysis, 2D annotation enrichment analysis was performed using Benjamini–Hochberg FDR Truncation with a 2% cutoff [47]. Pearson's correlation analysis was used to evaluate the correlation of individual protein amounts with MAPT. The significance of enrichment of proteins in Volcano plots was set from a fold change threshold of 1.5 (log₂FC ≥ 0.58) and significance set at an adjusted *P*-value ≤ 0.05. Adjustments for multiple comparisons were made using the Benjamini–Hochberg method. Student’s *t*-tests were used to evaluate differences in protein abundance between groups. Mitochondrial biosensor data were log-transformed, and multiple *t*-tests for control versus ePHF conditions at each time point were performed. Comparisons over full days were carried out with *t*-test on aggregated data. Synaptic Dye data were processed similar as these of mitochondrial sensor, except for the log transformation.

## Results

### ePHF-tau is cleared by astrocytes within two days in neuron-astrocyte 3D cultures

**Fig. 1:**
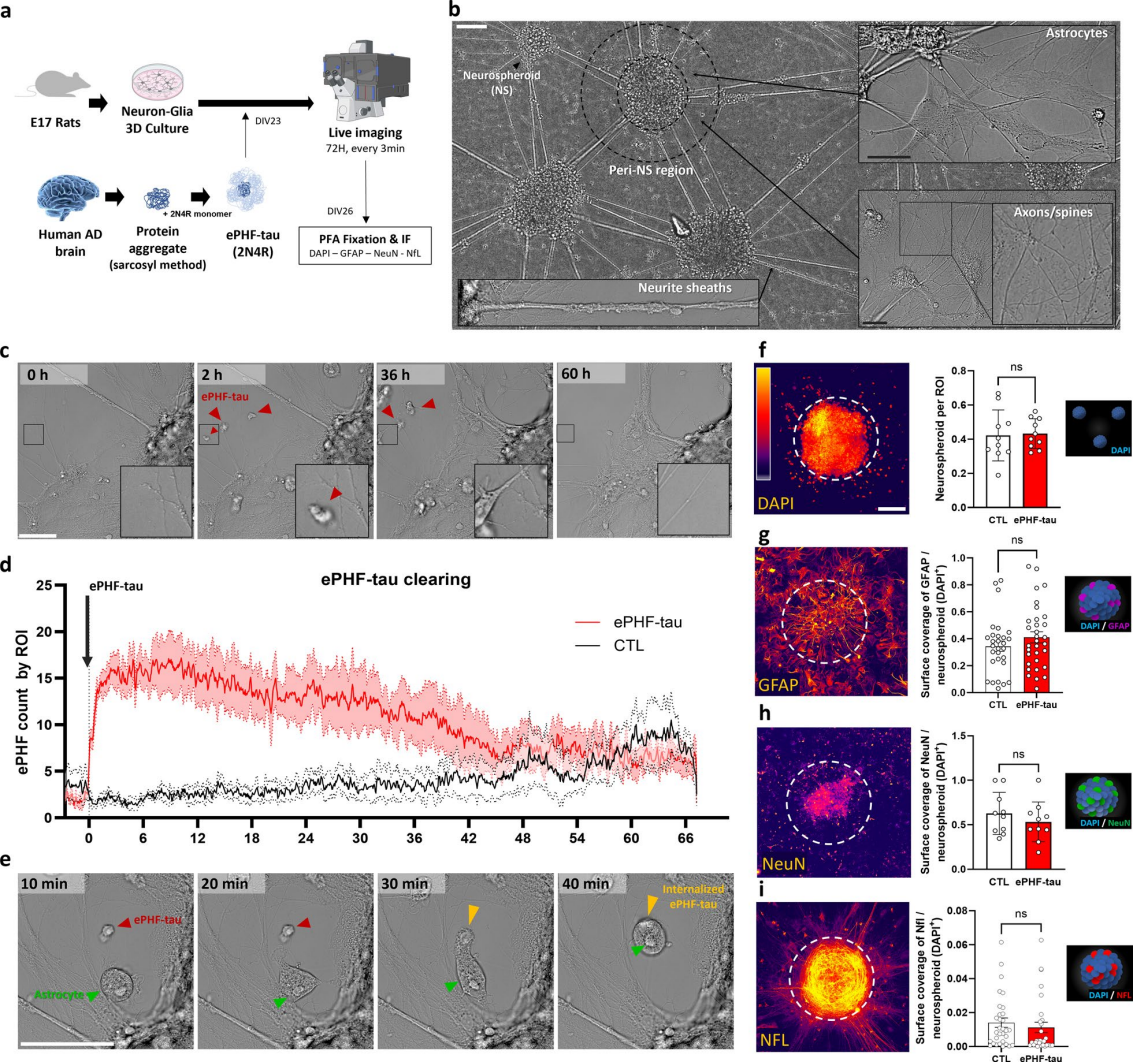
ePHF-tau is internalized by astrocytes in a neuron-glia 3D cell culture model. **a** Experimental setup: primary neuron-glia 3D co-cultures were treated with ePHF-tau, followed by live imaging for 72 h and post-fixation immunofluorescence (IF) for astrocytic (GFAP) and neuronal (NeuN, NFL) markers. **b** Overview of the neuron-glia 3D cultures showing neurospheroids (NS) and their surrounding microenvironment, including the peri-NS region and neurite sheaths. Scale bar: 100 µm. Examples at higher magnification highlight astrocytes (top right) and axonal/spine structures (bottom right). Scale bar: 10 µm. **c** Time-lapse series showing the gradual disappearance of ePHF-tau aggregates over 60 h. Red arrows point to the aggregates, with an inset providing a higher magnification of an aggregate being cleared at a specific location. Scale bar: 50 µm. **d** Time series quantifying ePHF-tau clearance per region of interest (ROI) in treated (red) versus control (DMSO, black) conditions, segmented using an AI-based model. Shaded areas represent SEM. **e** Time-lapse images of ePHF-tau uptake by astrocytes at different time points after aggregate addition (10, 20, 30, and 40 min). Red and yellow arrowheads indicate ePHF-tau aggregates, and green arrows point to astrocytes. Scale bar: 50 µm. **f** A confocal photograph of the neurospheroid positive for DAPI and the histogram represents the number of neurospheroids in the cultures. **g** A confocal photograph of astrocytic GFAP labelling in neuropsheroid and histogram presenting the GFAP surface by neuroscpheroide. **h** A confocal photograph of neuronal NeuN labelling in neuropsheroids and histogram showing NeuN surface by neuroscpheroid. **i** A confocal photograph of neuronal NFL labelling in neuropsheroids and histogram showing NFL surface by neuroscpheroide. (*n* = 3 cultures, 25 neurospheroids, 5 ROIs per condition;)

We used a 3D neuron-astrocyte co-culture embedded in matrigel to establish a robust in vitro model for studying effects of ePHF-tau. This 3D culture system described by Karahuseyinoglu et al. [60], better mimics the in vivo neural environment by promoting more physiologically relevant cellular organization, and providing accelerated electrical maturation and greater resilience to neurotoxic insults than 2D models [61] (Fig. 1a, b). To generate extracellular tau aggregates, we isolated PHF-tau from the sarkosyl-insoluble fraction of the frontal cortex of AD patients. The isolated PHF-tau was combined with recombinant monomeric 2N4R tau to produce PHF-tau(2N4R), referred to as ePHF-tau, following the protocol established by Courade et al. [32] (Fig. 1a). The cultures were treated with ePHF-tau and their response monitored over three days. The ePHF-tau aggregates sedimented rapidly and underwent progressive clearance over time (Fig. 1c). To quantify ePHF-tau clearing, we employed a machine learning segmentation model (Nikon.ai segment) to detect and track ePHF-tau aggregates in brightfield images, providing an estimation of their abundance. This analysis confirmed rapid sedimentation followed by efficient clearance over approximately two days (Fig. 1d). By manually analyzing the ROIs in the movies where the aggregates landed, we observed that astrocytes were involved in the clearance of ePHF-tau. These cells appeared in a flattened form, with highly active filopodia, and progressively and temporally circularized after invagination of the aggregate (Fig. 1e, Supplementary Movie). Following the live imaging period of three days, we fixed the cultures and performed immunofluorescence staining for various cell markers (Fig. 1f–i). The cell density (DAPI + , Fig. 1f), astrocytic reactivity (GFAP^+^, Fig. 1g), neuronal density (NeuN^+^, Fig. 1h), and neurofilament presence (NFL^+^, Fig. 1i) remained unchanged within the neurospheroids in response to ePHF-tau(2N4R) treatment, indicating no detectable impact on cell composition or structure.

### ePHF-tau promotes formation of synaptic vesicles and excitatory active zones

**Fig. 2:**
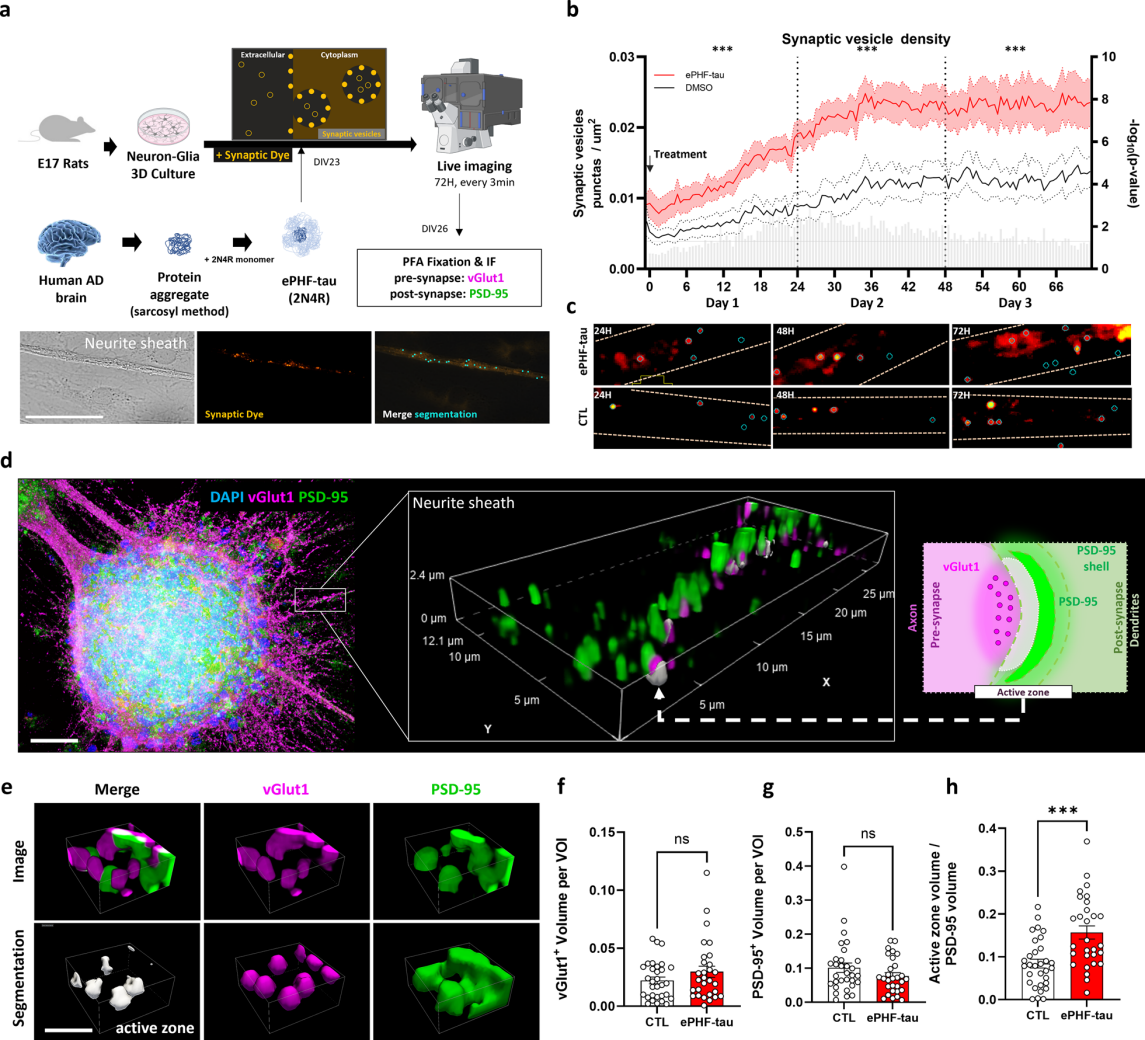
ePHF-tau induces modifications in synaptic markers in neuronal cultures. **a** Experimental design: treatment with ePHF-tau, live imaging over 72 h, followed by immunostaining for pre- (VGLUT1) and postsynaptic (PSD-95) markers. Bottom: brightfield and fluorescence images showing synaptic vesicle detection (light blue dots) in neurite sheaths. Scale bar: 50 µm. **b** Time series of synaptic vesicle density in control (black) and ePHF-tau-treated (red) conditions, relative to the time of treatment. Statistical comparisons for each time points (multiple *t*-tests) are shown as with gray bars on the right Y-axis as -log(*P*-value) and comparison over complete days 1, 2, and 3 are shown with asterisks (*) above the graph. **c** Fluorescence images of synaptic dye at 24, 48, and 72 h, with blue circles marking segmented synaptic vesicles in control and treated conditions. **d** Combined illustration and methodological overview of neurospheroid immunostaining analysis. The inset shows an example of a 3D volume of interest (VOI) analyzed for synaptic markers. The schematic on the right illustrates the approach used for segmenting active zones and postsynaptic compartments. Scale bar: 100 µm. **e** 3D visualization of the active zone (gray) along with VGLUT1 (magenta) and PSD-95 (green) in segmented regions of interest (ROI). Scale bar: 5 µm. **f–h** Quantification of total VGLUT1 volume (**f**), total PSD-95 volume (**g**), and active zone volume normalized to PSD-95 surface area (**h**) in PERI-NS sheath (*n* = 3 cultures, 25 neurospheroids, 5 ROIs per condition; ***P < 0.001)

Numerous recent studies suggest that extracellular tau or amyloid could play a role in cerebral excitotoxicity by disrupting the synaptic and neuronal balance [62–65]. To evaluate the effect of ePHF-tau on synaptic equilibrium, cultures were stained with the synaptic dye BioTracker, which is a fluorescent cationic styryl dye that monitors synaptic activity at neuromuscular junctions or synapses [66]. ROIs containing dendritic segments near neurospheroids (identified through Brightfield imaging) were monitored over three days (Fig. 2a, Fig. S1). We quantified the number of synaptic dye puncta within the various ROIs. In control cultures, a slight progressive increase in the number of synaptic vesicles was observed over time in these regions. However, in cultures treated with ePHF-tau, this increase was significantly accelerated from the first day post-treatment, suggesting that ePHF-tau markedly enhances synaptic vesicle production, which may indicate an excitotoxic response (Fig. 2b, c). To deepen our conclusions, we performed dual immunofluorescence for the main excitatory synaptic proteins, specifically the postsynaptic protein PSD95 and the presynaptic protein VGLUT1, at 3 days after treatment (Fig. 2d). Treatment with ePHF-tau did not affect the total number of VGLUT1 puncta (Fig. 2f) or the volume of PSD95 (Fig. 2g). However, we observed a significant increase in the number of active zones (represented by the overlap between VGLUT1 and PSD95) in cultures treated with ePHF-tau (Fig. 2h). This suggests that treatment with ePHF-tau alone could promote the formation of synaptic vesicles in neuronal dendrites and potentially increase the number of excitatory active zones between neurons.

### Impact of ePHF-tau on synaptosomes and mitochondria: involvement of astrocytic proteins

**Fig. 3:**
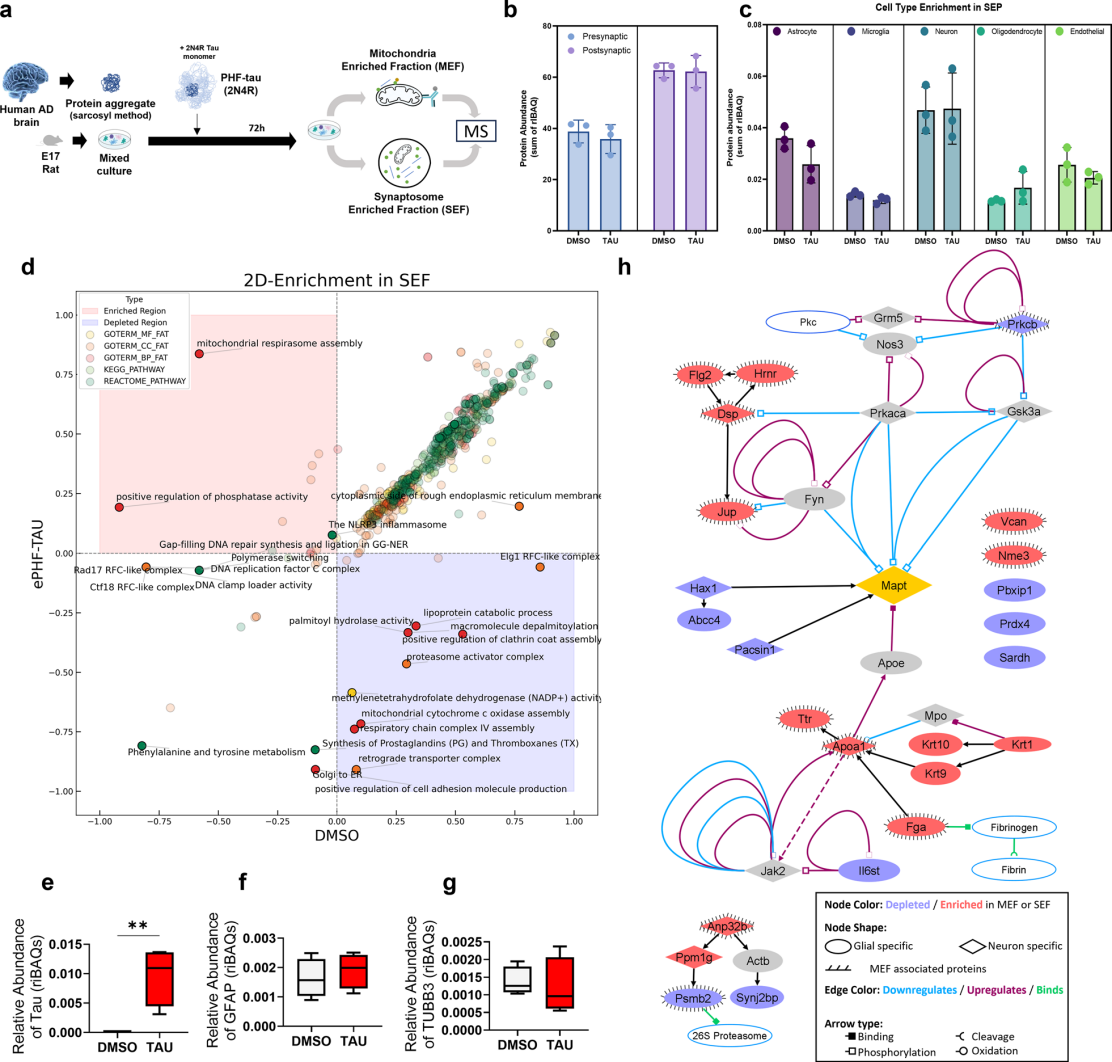
Proteomic analysis of synaptosome and mitochondrial fractions reveals differential protein regulation under ePHF-tau treatment. **a** Experimental workflow to investigate the interactions of ePHF-tau in a rat primary mixed culture with 72-h ePHF-tau treatment, followed by isolation of synaptosome-enriched fractions (SEF; *n* = 3 for DMSO, *n* = 3 for ePHF-tau) and mitochondria-enriched fractions (MEF; *n* = 4 for DMSO, *n* = 4 for ePHF-tau) for proteomic analysis. **b** Quality check of SEF fractions: Synaptic marker enrichment (presynaptic and postsynaptic proteins) in DMSO and tau-treated conditions, confirming the expected protein content in SEF fractions. **c** Quality check of SEF fractions based on cell-type enrichment markers (astrocyte, microglia, neuron, oligodendrocyte, and endothelial) showing representation of different cell types within the synaptosome fractions under DMSO and tau-treated conditions. **d** Differentially expressed functional annotations (2D enrichment) in SEF. Enriched terms are shown in red region, and depleted terms are shown in blue region. Dots colors indicate the annotation types. **e**–**g** Box plots comparing the relative abundance of MAPT (**e**), GFAP (**f**), and TUBB3 (**g**) between DMSO and ePHF-tau conditions, showing differential regulation of these proteins in the MEF. **h** Protein–Protein interaction network of the modified proteins in SEF/MEF

To explore the intracellular effects of ePHF-tau, we treated cultures with ePHF-tau and isolated SEF and MEF after three days. These fractions were subsequently prepared for proteomic analysis via mass spectrometry (Fig. 3a). Each identified protein was annotated using synaptic proteome databases [67], brain cell-specific proteomes [68], and mitochondrial proteomes [69]. The quality of isolation was confirmed by comparing the enrichment of synaptosome- or mitochondria-associated proteins between fractions (Fig. S2).

Our analysis showed that the SEF contained approximately 40% presynaptic and 60% postsynaptic proteins. These proportions did not significantly change following ePHF-tau treatment (Fig. 3b). Similarly, the SEF composition remained primarily neuronal and astrocytic, with minor microglial and oligodendrocytic protein presence, unaffected by ePHF-tau exposure (Fig. 3c). These results indicate that ePHF-tau treatment does not globally alter the synaptosomal protein distribution or the astrocytic and glial population within the synaptic compartment.

Among proteins enriched in SEF, the tau protein was significantly increased in synaptosomes with ePHF-tau treatment, suggesting that tau could enter synaptic compartments (Fig. S3, Table S1). Several significantly altered proteins in the SEF, such as Synj2bp, Pacsin1, and Pbxip1, are associated with mitochondrial functions and regulatory mechanisms (Fig. S3, Table S1). Functional and ontological enrichment analysis supported these findings, revealing a strong association with mitochondrial properties, including mitochondrial respiration, cytochrome *c* activity, and respiratory chain complexes, as well as phosphatase activity regulation (Fig. 3d). Other significantly altered proteins in the SEF are involved in signaling pathways related to phosphatase activity, proteasomal function, and cell adhesion. This suggests that extracellular tau can influence mitochondrial function in the synaptic compartments (Fig. 3d).

Then, we isolated mitochondrial fraction for proteomic analysis. We confirmed significant enrichment of mitochondrial proteins in the MEF compared to SEF (Fig. S2). MEF showed a strong association with tau protein (Fig. 3e, Fig. S3, Table S2), in contrast to other cytoskeletal proteins like GFAP (Fig. 3f) and Tubb3 (Fig. 3g). These results imply that tau may penetrate cells and become closely associated with mitochondria. Interestingly, several proteins were significantly enriched in the MEF following ePHF-tau treatment, including Vcan, Hrnr, Krt1, Flg2, Ttr, Krt9, Dsp, Fga, Nme3, Apoa1, Anp32b, Krt10, and Jup. Conversely, proteins such as Prkcb, Sardh, and Psmb2 were notably reduced (Fig. S3, Table S2). The network of interaction of the enriched or depleted proteins in SEF and/or MEF (Fig. 3h) highlighted a central position of tau. Intriguingly, mitochondrial-associated proteins related to hypoxia, oxidoreductase activity, and nucleoside phosphatase binding, were predominantly astrocytic. Together, these results suggest that ePHF-tau may preferentially affect the mitochondrial system of glial cells (Tables S1 and S2).

### Long-term live imaging reveals divergent mitochondrial responses in neurons and astrocytes under ePHF-tau exposure

**Fig. 4:**
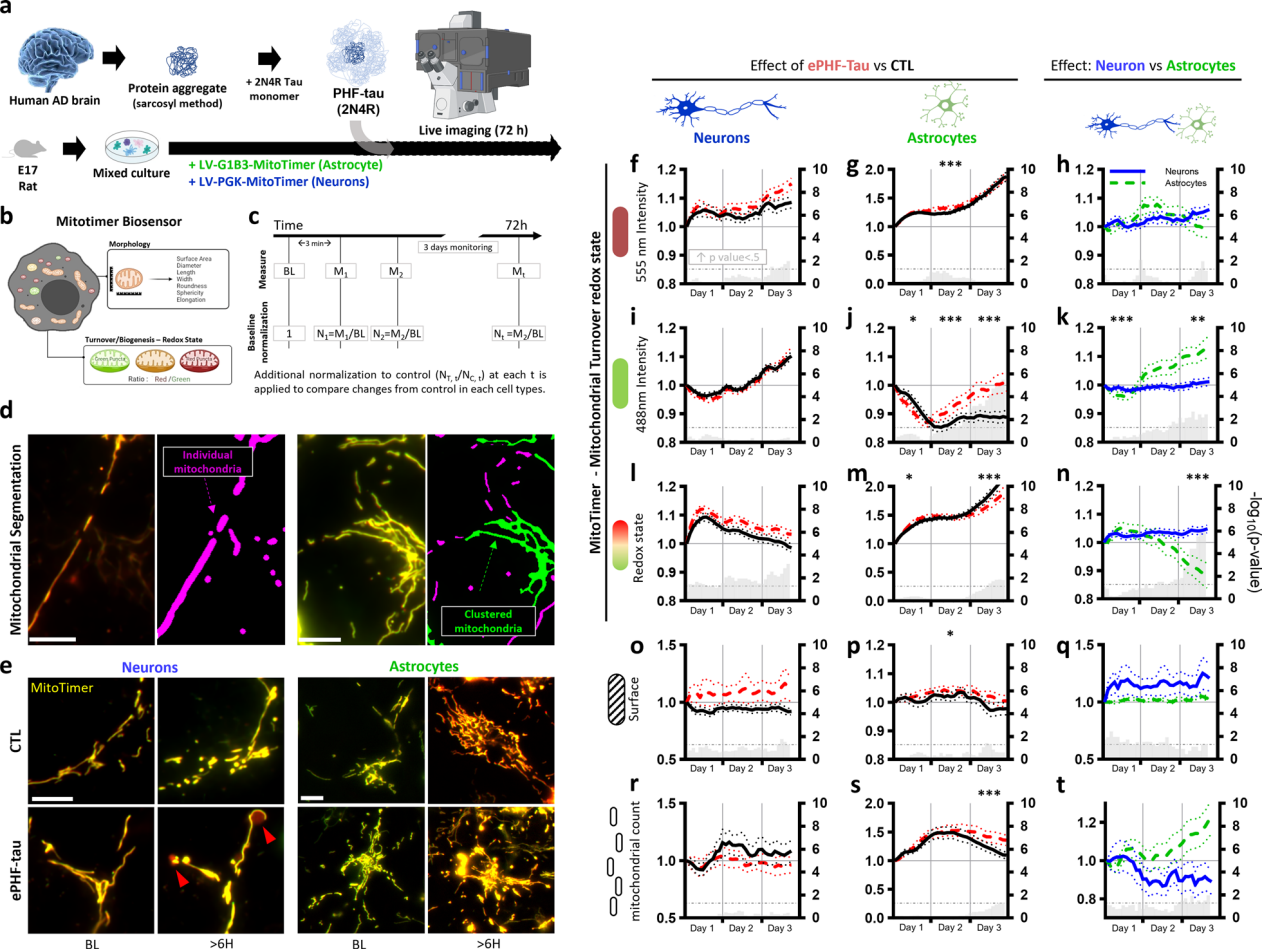
ePHF-tau negatively affects mitochondrial turnover in neurites. **a** Experimental design: primary mixed cultures transduced with mitochondrial sensors (MitoTimer) were imaged over 72 h in the presence of ePHF-tau. **b** Diagram of the MitoTimer biosensor measuring mitochondrial morphology and turnover/redox state. **c** Data normalization process: mitochondrial features were normalized to baseline (BL) and then compared to controls. **d** Mitochondrial segmentation illustrating the focus on individual mitochondria (purple) versus clusters (green) in neurons (left) and astrocytes (right). Scale bar: 10 µm. **e** Representative Mitotimer fluorescence images in neuronal neurites (left) and astrocytic processes (right) at baseline and > 60 h after ePHF-tau treatment. Red arrows indicate swollen, red mitochondria. Scale bar: 10 µm. **f–t** Comparative analysis of mitochondrial features in and between neurons and astrocytes. Each row represents a specific feature: fluorescence intensity at 555 nm (**f**, **g**, **h**), 488 nm (**i**, **j**, **k**), redox state (**l**, **m**, **n**), mitochondrial surface area (**o**, **p**, **q**), and mitochondrial count (**r**, **s**, **t**). The left columns (**f**, **i**, **l**, **o**, **r**) show the effects of ePHF-tau treatment (red lines) versus control (black lines) in neurons, the middle columns (**g**, **j**, **m**, **p**, **s**) depict the same comparison in astrocytes, and the right columns (**h**, **k**, **n**, **q**, **t**) compare neurons (blue lines) with astrocytes (green lines) relative to their respective controls. Gray bars in each graph represent -log(*P*-value) for comparisons at each time point on the right y-axis. Asterisks above the graphs indicate *P*-values for comparisons over entire days (means and SEM, *P < 0.05, **P < 0.01, ***P < 0.001, ****P < 0.0001)

To investigate the dynamic effects of ePHF-tau on mitochondria in neurons and astrocytes, we transduced primary mixed cultures with MitoTimer, a mitochondrial biosensor. Live imaging was performed over 72 h post-ePHF-tau treatment using advanced fluorescence microscopy equipped for high-throughput acquisition and analysis (Fig. 4a, b) [29, 30]. Mitochondrial feature measurements made with 2-h intervals were normalized to baseline measurements obtained at the beginning of the experiment (Fig. 4c) and individual mitochondria were segmented, excluding clustered mitochondria (Fig. 4d) [30, 34]. Representative images from MitoTimer fluorescence revealed distinct mitochondrial behavior in neurons and astrocytes at baseline and after more than 60 h of ePHF-tau exposure (Fig. 4e). Data were compared at each time point (transformed* P*-values displayed as bars in Fig. 4f–t), and over entire days (*P*-values represented as stars in Fig. S4).

In neurons, ePHF-tau treatment induced minor mitochondrial changes. While there was a slight increase in the redox state (indicated by a shift toward red Mitotimer fluorescence (555 nm) reflecting oxidation), these changes were not statistically significant across all tests (Fig. 4f, i, l). Mitochondrial turnover, measured via 555 nm and 488 nm fluorescence, showed trends toward increased oxidation and stress, but these effects remained limited and did not result in significant dysfunction. Notably, the primary driver of oxidation was the rise of oxidized complexes (red (555 nm) Mitotimer signal) rather than a decrease in biogenesis (green (488 nm) Mitotimer signal). This observation was supported by significant increases of neuronal mitochondrial ATP after 15 h (Fig. S5). In terms of morphology, neuronal mitochondria exhibited some oxidative stress and swelling. The mitochondrial surface area in neurons exhibited a modest increase over time, with a transient significant elevation observed at the end of 72 h (Fig. 4o). However, this change did not remain statistically significant throughout the experiment. Concurrently, the number of individual mitochondria showed a slight reduction. However, this decrease did not reach statistical significance (Fig. 4r). These findings suggest that ePHF-tau exerts a minimal impact on mitochondrial morphology in neurons, reflected by only minor and non-significant changes in both surface area and mitochondrial count.

In contrast, astrocytic mitochondria exhibited a more pronounced and significant response to ePHF-tau exposure. During the first 36 h, mitochondrial redox states and turnover remained relatively stable (Fig. 4g, j, m). However, after this period, alterations emerged. Astrocytes demonstrated increased mitochondrial turnover, as evidenced by the rise in green (488 nm) fluorescence (Fig. 4j), reflecting heightened mitochondrial biogenesis. This increase in mitochondrial activity and turnover, combined with changes in the redox state (Fig. 4m), indicates that astrocytes are more affected by ePHF-tau over time, with a more adaptive response compared to the limited changes observed in neurons. In astrocytes, mitochondrial surface area exhibited a slight but significant increase over time (Fig. 4p). More notably, the number of individual mitochondria steadily increased from the 36-h mark onward, suggesting a sustained rise in mitochondrial biomass (Fig. 4s). This observation aligns with the increase in green (488 nm) Mitotimer fluorescence indicative of increased mitochondrial biogenesis. Together, these results suggest that astrocytes undergo a significant increase in mitochondrial biogenesis in response to ePHF-tau treatment.

Comparison between neurons and astrocytes highlighted significant differences in how these cell types manage mitochondrial stress. Astrocytes showed a delayed but robust response characterized by increased mitochondrial turnover and shifts in the redox state, whereas neurons exhibited only minor oxidative changes, which were not statistically significant across all tests (Fig. 4h, k, n, q, t). Together, we observed divergences regarding the direction of the redox state/turnover modification and the main driver that mediated that change (green component (488 nm) in astrocytes, red component (555 nm) in neurons). These findings suggest that ePHF-tau has a primary impact on astrocytes where it drives significant changes in mitochondrial turnover and biogenesis, while inducing limited mitochondrial stress in neurons. This indicates that astrocytes play a crucial role in the cellular response to ePHF-tau, potentially adapting their mitochondrial function to mitigate long-term damage caused by the aggregates.

### Accumulation of tau 4R in astrocytes stimulates mitochondrial biogenesis and alters synaptic plasticity

**Fig. 5:**
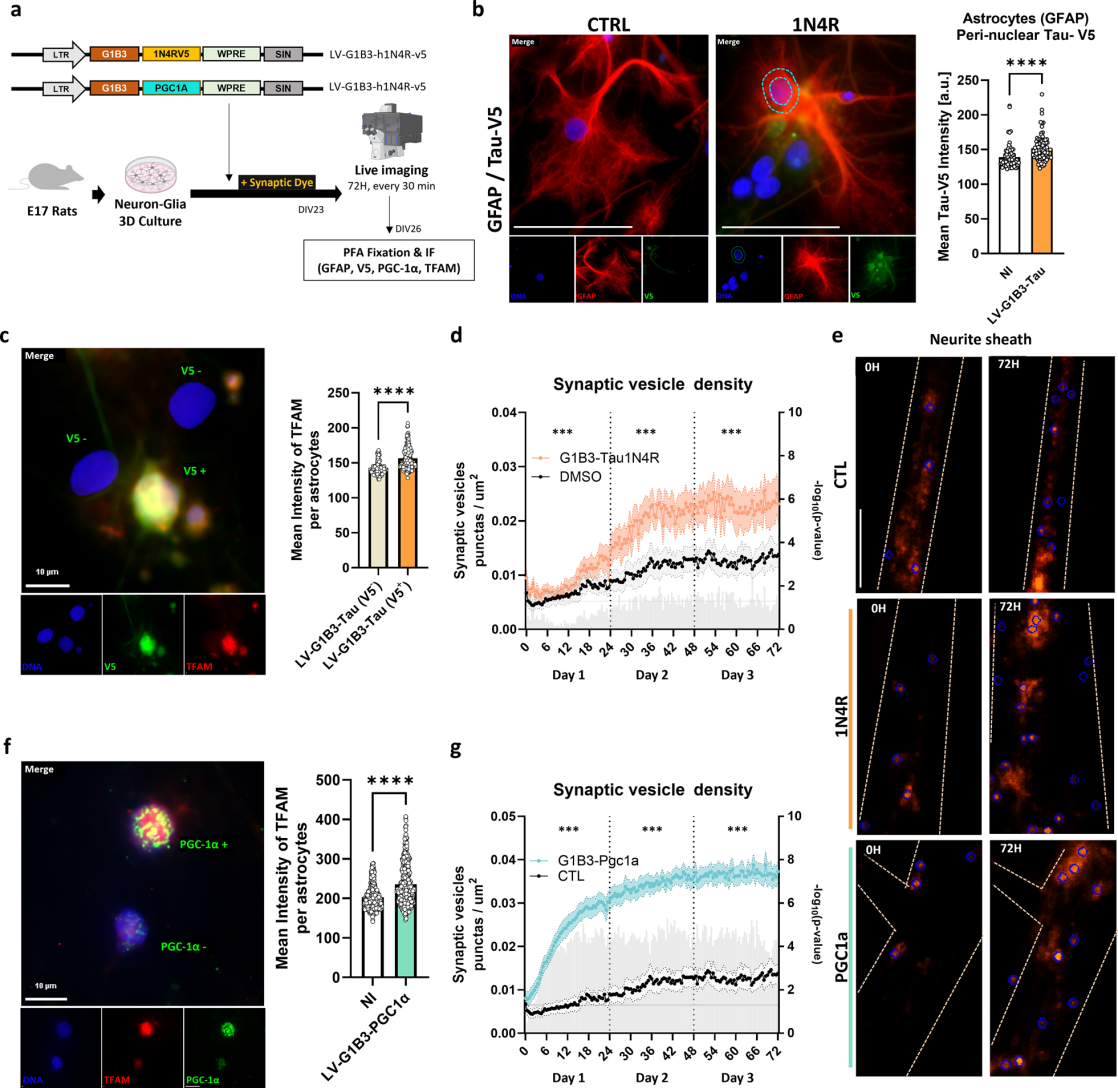
Astrocytic modifications alone enhance synaptic vesicle density in neurons. **a** Experimental design: primary mixed cultures with astrocytes infected to overexpress WT human Tau-1N4R-V5 or PGC-1α were stained with a synaptic dye and imaged over 72 h in the presence of ePHF-tau. **b** Immunofluorescence images of astrocytes (GFAP) stained for Tau-V5 tag after 72 h treatment. Quantification of Tau-V5 intensity in peri-nuclear space of GFAP-positive cells is shown on the right. Scale bar, 50 μm; dashed blue line: peri-nuclear space. **c** Microscopic images of cultures infected with G1B3-Tau-1N4R-V5 and stained for V5 (green) and TFAM (red). Cells can be positive or negative for V5 expression (as displayed by text on merge image). Right, a bar plot of the quantification of the mean TFAM intensities in nucleus. **d** Time series of synaptic vesicle density in neurons co-cultured with G1B3-Tau-1N4R-V5 astrocytes (orange) versus DMSO-treated controls (black). Statistical comparisons for each time point (multiple *t*-tests) are shown as gray bar on the right Y-axis as -log(*P*-value) and statistical comparison for data subsets aggregated by day is shown with asterisks (*) above the graph. **e** Fluorescence images of synaptic dye at baseline and 72 h in control, 1N4R, and PGC-1α conditions, with blue circles marking segmented synaptic vesicle. Scale bar, 10 μm. **f** Same as (**c**) but with G1B3-PGC-1α infection of astrocytes. **g** Same as (**d**), with G1B3-PGC-1α infection of astrocytes. *P < 0.05, **P < 0.01, ***P < 0.001

At this stage, our results suggest that PHF-tau internalized by astrocytes activates their mitochondrial biogenesis, which may be responsible for the observed modifications in surrounding synapses. To understand whether the intracellular presence of the tau 4R protein in astrocytes alone can stimulate this mitochondrial biogenesis and induce synaptic consequences, we specifically overexpressed the human 4R tau form in astrocytes from mixed cultures and monitored synaptic vesicle formation by live imaging over three days (Fig. 5a). As previously demonstrated, this viral construct induced tau accumulation exclusively in astrocytes (Fig. 5b) without significant alterations in morphology [29]. Immunohistochemistry performed three days post-infection shows a significant increase in nuclear levels of the transcription factor TFAM in astrocytes expressing tau 4R (V5^+^) (Fig. 5c). Real-time imaging of these astrocytes revealed that this progressive accumulation of tau 4R was accompanied by a significant increase in synaptic vesicle densities compared to that on day 1 (Fig. 5d, e). These results suggest that the accumulation of tau 4R in astrocytes induces an increase in mitochondrial biogenesis within these cells, which may explain a synaptic excitotoxicity.

To demonstrate that the increase of mitochondrial biogenesis in astrocytes alone could lead to an increase in synaptic vesicles, we used another construct allowing the overexpression of PGC-1α specifically in astrocytes. PGC-1α is a crucial transcription factor for mitochondrial biogenesis in astrocytes [33, 70, 71]. We found that overexpression of PGC-1α induced an increase in mitochondrial biogenesis within three days, manifested as elevated levels of TFAM in the nuclear region of cells with nuclei positive for PGC-1α (Fig. 5f), replicating the effects observed with tau (Fig. 5c). Interestingly, synaptic dye analysis revealed that the activation of mitochondrial biogenesis by PGC-1α overexpression quickly led to a significant increase in the density of synaptic vesicles (Fig. 5e, g). These results validate the hypothesis that the internalization of extracellular tau by astrocytes can directly influence their mitochondrial biogenesis, thus affecting surrounding synaptic function.

## Discussion

The primary objective of this study was to explore the impact of extracellular tau aggregates on neuronal and astrocytic cell populations. Specifically, we aimed to understand how these aggregates influence mitochondrial responses and associated synaptic imbalances while attempting to highlight the potential underlying mechanisms of these disturbances. Our findings emphasize the central role of tau aggregates in disrupting synaptic functions. While many studies have focused on hyperphosphorylated forms of tau to explain synaptic toxicity, our work demonstrates that even non-hyperphosphorylated tau aggregates (ePHF-tau) can induce notable and rapid synaptic dysfunctions, primarily through their effects on astrocytes. This challenges the notion that only neurons are affected by different tau forms and opens numerous perspectives for understanding the mechanisms underlying tau-associated cognitive disorders. Additionally, the association of ePHF-tau with mitochondrial disturbances highlights potential early targets of tau pathology, underscoring the importance of considering therapeutic approaches that address tau aggregates and the associated mitochondrial consequences within specific subpopulations.

### Impact of extracellular tau on synaptic function

Numerous studies have demonstrated how extracellular tau disrupts synaptic function. For example, in animals, soluble aggregates of tau can inhibit long-term depression (LTD) in the dorsal hippocampus, thereby disrupting the regulation of synaptic strength and the modeling of neuronal circuits, as shown by Kimura and colleagues [72]. Other work has shown that the uptake of oligomeric tau by neurons can trigger abnormal accumulation of intracellular tau, which disrupts fast axonal transport and compromises overall neuronal homeostasis [73]. Additional studies indicate that the injection of N-terminal fragments of extracellular tau into the brain induces changes in synaptic activity, independent of signs of neurodegeneration [74].

Due to the pleiotropic nature of the tau protein, several explanations have been proposed in the literature to describe the toxic effect of extracellular tau, such as a direct action of tau on membrane receptors [75]. Indeed, tau-induced alterations of NMDA and AMPA receptors lead to uncontrolled calcium currents, exacerbating excitotoxic phenomena and thus contributing to the observed synaptic imbalances [76, 77]. Extracellular tau can also play a crucial role in the regulation of synaptic signaling, notably via muscarinic acetylcholine receptors. tau binds to these receptors with a much higher affinity than acetylcholine, thus disrupting inter-neuronal transmission [78]. This interaction is exacerbated by the presence of oligomeric tau, which degrades the morphology and the density of dendritic spines and increases intracellular levels of ROS and calcium, thus contributing to neurotoxicity [63, 74, 79].

Our results confirm and extend these observations. We found that ePHF-tau, even in a non-hyperphosphorylated form, significantly impacted synaptic dynamics without affecting the overall structure of the cells. In our 3D co-culture model of neurons and astrocytes, ePHF-tau induced a rapid increase in the production of synaptic vesicles and excitatory active zones, suggesting an excitotoxic effect. Although there were no alterations in neuronal density, neuroinflammation, and structure of neurospheroids during the three days, the increased overlap of VGLUT1 (presynaptic) and PSD95 (postsynaptic) after treatment revealed strengthening of synaptic active zones. This observation was corroborated by mass spectrometry analyses of synaptosomal fractions, which did not indicate overall modifications of pre- and postsynaptic proteins or major changes in cell subtypes. However, more subtle alterations in signaling pathways, notably those involved in phosphatase, protease and mitochondrial metabolic activities, were detected, aligning with the conclusions of previous studies [74, 80]. These results suggest that tau has entered synapses and disrupted specific signaling pathways related to its degradation and post-translational processing. Furthermore, the enrichment of mitochondrial terms in SEF reflects the role of mitochondria in neuronal energy homeostasis and calcium buffering to fulfil the energy demands [81, 82] of increased synaptic density due to extracellular tau. In sum, these results underline that the toxic effect of extracellular tau does not solely depend on its phosphorylation state but results from the complexity of the synaptic interactions it disrupts and the structural alterations it provokes. In addition, the predominant changes in neuron- and astrocyte-specific genes expand the research focus beyond being just neuron-centric, broadening the horizons for studies in extracellular Tau and its effect on other cell types [19, 83]. All these modifications confirm that pathological tau profoundly disrupts the excitation–inhibition balance, which is directly linked to cognitive deficits in tauopathies [77, 84].

### Mitochondrial dysfunction induced by extracellular tau: importance of astrocytes

Previous studies have demonstrated that extracellular tau can induce various mitochondrial dysfunctions. By inhibiting mitochondrial calcium efflux via the Na⁺/Ca^2^⁺ exchanger, tau causes mitochondrial depolarization, making neurons and astrocytes more vulnerable to calcium-induced cell death [13, 14]. Caspase-cleaved tau disrupts mitochondrial transport by impairing TRAK2 function, fragmenting mitochondria, and decreasing ATP production [85]. Furthermore, tau cleaved by caspase-3 increases mitochondrial fragmentation and depolarization while elevating ROS levels—effects that can be attenuated by Nrf2 activation [86]. Proteomic analysis also revealed that the astrocyte-specific proteasome family member Psmb2 was significantly depleted due to elevated tau levels, which reflects the inability of proteasomes to degrade tau protein in effect of heightened ROS levels. The insoluble tau aggregates modify membrane ion conductance and activate calcium channels, leading to calcium influx and ROS production via NADPH oxidase, ultimately resulting in neuronal death [14]. Moreover, tau oligomers hinder fast axonal transport, causing an accumulation of intracellular tau and blocking the transport of organelles along microtubules [87, 88].

Although these mechanisms are well described in neurons, the consequences on astrocytes have been suggested much more recently. Recent evidence emphasizes that astrocytes are not merely passive players but are actively involved in propagating tau pathology [19, 89, 90]. Astrocytes play an essential role in the neurotoxic/synaptotoxic effects of tau protein through reduced availability of gliotransmitters, thus contributing to tau pathology in AD [91, 92]. Extracellular tau oligomers compromise the ability of astrocytes to manage extracellular glutamate, thereby exacerbating excitotoxicity [93, 94]. The tau oligomers also disrupt intracellular calcium signaling and gliotransmitter processing, affecting synaptic transmission [94, 95]. Additionally, exposure of astrocytes to extracellular tau can induce cellular senescence, characterized by a senescence-associated secretory phenotype and increased neuronal toxicity [96]. Here, we report an upregulation of Apoa1 in MEF which is known to positively regulate Apoe, another tau protein interactor. Furthermore, we found that Apoa1 interacts with Ttr, which has neuroprotective abilities like amyloid clearance in physiological conditions [97] and is considered a prospective biomarker for AD [98, 99].

In our study, using a viral approach specific to neurons and astrocytes, we analyzed mitochondrial turnover, redox state, and morphology over 72 consecutive hours following treatment with ePHF-tau. Our findings reveal that astrocytes exhibit a more substantial mitochondrial response compared to neurons when exposed to ePHF-tau. There is a significant increase in mitochondrial turnover and mass, suggesting an adaptive response to the mitochondrial stress induced by tau. However, these changes manifest later compared to the immediate and acute effects observed in neurons, indicating that astrocytes are initially more resilient but later succumb to stress.

The increase in mitochondrial biogenesis in astrocytes indicates an attempt to adapt to mitochondrial stress. This is in line with prior studies showing that astrocytes can engage in mitochondrial reorganization and biogenesis as a protective response [30]. This behavior contrasts with that of neurons, which show signs of oxidative stress without activating compensatory mechanisms. Accordingly, our proteomic data present a depletion of astrocyte specific proteins related to protein degradation (Psmb2), metabolism of amino acids (Sardh), and neuron specific glutamate binding (Prkcb) which is involved in synaptic plasticity. To delve deeper into this phenomenon, we induced tau accumulation in astrocytes, as well as an increase in mitochondrial biogenesis via PGC1α overexpression and reproduced the response to ePHF-tau. Our results suggest that astrocytic mitochondrial adaptations may play a crucial role in moderating neuronal dysfunction.

These results reinforce the idea that astrocytes actively manage tau-related toxicity, playing a crucial role in tauopathies as supporting cells and active players [29, 30]. Astrocytes can capture, internalize, and sometimes transmit these aggregates, thereby aggravating the pathology [14, 19, 100]. Tau accumulation leads to phenotypic changes to a neuroinflammatory profile, compromising their neuron-supporting function [19, 101, 102]. This disrupts essential functions of astrocytes in maintaining ionic homeostasis, energy supply, and synaptic balance [29, 83, 103], while the release of pro-inflammatory molecules and stress factors by dysfunctional astrocytes increases neuronal vulnerability to degeneration [104, 105].

### Impact of extracellular accumulation of aggregated tau on brain development

In this in vitro study, our results highlight plausible links between extracellular accumulation of aggregated tau and alterations of astrocyte functioning and neuronal development. It is well established that during critical stages of neuronal maturation, the astrocytes play an essential role in the formation of brain circuits [106]. Astrocytes regulate synaptic homeostasis, modulate neuronal transmission, and support key metabolic processes and neurogenesis [107, 108] mainly due to their mitochondrial functions, which are essential for meeting the energy needs of developing neurons [109, 110]. Recent studies have demonstrated that astrocytic mitochondrial dysfunctions can profoundly disrupt neuronal signaling and synaptic plasticity, highlighting their central role in brain maturation [33, 111, 112].

In the present study, ePHF-tau disproportionately impacted the mitochondrial system of astrocytes, potentially compromising their supportive functions in neuronal development. For example, although the increase in mitochondrial biogenesis may seem like a compensatory mechanism in response to the presence of extracellular tau, it could reduce the efficiency of astrocytes in regulating neurotransmitter reuptake and releasing trophic factors essential for neuronal development [113, 114].

We also found that tau 1N4R accumulation induced an increase in TFAM in astrocytes, suggesting that increased tau in astrocytes improves mitochondrial biogenesis. Overexpression of PGC-1α in astrocytes led to increased synaptic vesicles in cultures, suggesting that disturbances in the mitochondrial dynamics in astrocytes could have lasting impacts on brain maturation. Interestingly, research has revealed the presence of hyperphosphorylated tau aggregates in the brains of young children, precisely when astrocytes begin to differentiate and integrate into neuronal circuits [115]. Certain developmental diseases, such as Down syndrome, exhibit anomalies of hyperphosphorylated and aggregated tau [116, 117]. This raises the hypothesis that early disturbances in the phosphorylation or aggregation of the tau protein could contribute to complex neurodevelopmental anomalies, such as autism or attention-deficit/hyperactivity disorder, where imbalances between excitation and inhibition are frequently reported [118, 119]. These observations underscore the importance of further exploring the role of astrocytes and their mitochondria in neuronal maturation to better understand the underlying mechanisms of neurodevelopmental disorders associated with tau dysfunction.

## Conclusion

This study highlights the significant impact of extracellular aggregates of tau, even in a non-hyperphosphorylated form (ePHF-tau), on the synaptic and mitochondrial functions of neurons and astrocytes. Our results demonstrate that ePHF-Tau induces rapid synaptic dysfunction, primarily through astrocytes, challenging the notion that only hyperphosphorylated forms of tau are toxic and that neurons are the sole cell type affected. We observed that ePHF-tau disrupts synaptic dynamics by increasing the production of synaptic vesicles and strengthening excitatory active zones, suggesting an excitotoxic effect. Moreover, ePHF-tau causes mitochondrial dysfunction, particularly in astrocytes, which respond with increased mitochondrial biogenesis. Although this mechanism may seem compensatory, it could compromise the supportive functions of astrocytes, such as regulating neurotransmitter reuptake and releasing trophic factors essential for neuronal development. These mitochondrial and synaptic disturbances could have lasting impacts on brain connectivity. In summary, our study highlights astrocytes as supporting cells and active players in tau-related pathology. Pursuing further research to better understand the underlying mechanisms of these dysfunctions is crucial. This could open new therapeutic perspectives targeting both tau aggregates and the associated mitochondrial consequences within specific cell subpopulations.

## Supplementary Information

**Additional file 1. Supplementary Material & Methods. Figure S1.** Analysis of synaptic vesicle density and cell body exclusion. **Figure S2.** Enrichments in synaptosomal or mitochondrial proteins in SEF and MEF. **Figure S3.** Volcano plots for SEF and MEF fractions. **Figure S4.** Mitochondrial features quantifications pooled by days. **Figure S5.** ATP monitoring in neuron after ePHF-tau. **Table S1.** Significantly enriched and depleted proteins in SEF. **Table S2.** Significantly enriched and depleted proteins in MEF.

**Additional file 2.** Time-lapse brightfield microscopy of neurospheroid edge dynamics captured at 40× magnification over a 3-day period with 4-min intervals between frames. The sequence shows exogenously added cellular aggregates at the neurospheroid periphery and their subsequent internalization by an astrocyte.

## Data Availability

All data generated or analyzed during this study are included in this article and Additional file or are available from the corresponding author on reasonable request.

## Abbreviations

AD: Alzheimer's disease
PHF-tau: Paired helical filaments tau
ePHF-tau: Extracellular paired helical filaments tau
DIV: Days in vitro
DMSO: Dimethyl sulfoxide
BSA: Bovine serum albumin
WPRE: Woodchuck hepatitis virus postregulatory element
cPPT: Central Polypurine Tract
NeuN: Neuronal nuclei
GFAP: Glial fibrillary acidic protein
DAPI: 4',6-Diamidino-2-phenylindole (nuclear stain)
ROI: Region of interest
MEF: Mitochondrial-enriched Fraction
IBAQ: Intensity-based absolute quantification
LFQ: Label-free quantification
DAVID: Database for Annotation, Visualization, and Integrated Discovery
