# Supplementary material for: Extracellular PHF-tau modulates astrocyte mitochondrial dynamics and mediates neuronal connectivity
Source: Transl Neurodegener. 2025 Mar 24;14:13. doi: 10.1186/s40035-025-00474-9 (PMC11931834; doi:10.1186/s40035-025-00474-9)
Supplement: Supplementary file 1 — Additional file 1. Supplementary Material & Methods. Figure S1. Analysis of synaptic vesicle density and cell body exclusion. Figure S2. Enrichments in synaptosomal or mitochondrial proteins in SEF and MEF. Figure S3. Volcano plots for SEF and MEF fractions. Figure S4. Mitochondrial features quantifications pooled by days. Figure S5. ATP monitoring in neuron after ePHF-tau. Table S1. Significantly enriched and depleted proteins in SEF. Table S2. Significantly enriched and depleted proteins in MEF. [file 40035_2025_474_MOESM1_ESM.pdf]

Supplementary material & methods

Synaptosomes proteomic mass spectrometry

Protein digestion: Synaptosomal fractions were digested according to a modified version of the iST method 80 (named miST method). Briefly, 50 µl solution of PBS was supplemented with 50 µl miST lysis buffer (1% sodium deoxycholate, 100 mM Tris pH 8.6, 10 mM DTT) and heated at 95°C for 5 min. Samples were then diluted 1:1 (v:v) with water, and reduced disulfides were alkylated by adding ¼ vol of 160 mM chloroacetamide (final 32 mM) and incubating at 25°C for 45 min in the dark. Samples were adjusted to 3 mM EDTA and digested with 0.5 µg Trypsin/LysC mix (Promega #V5073) for 1h at 37°C, followed by a second 1h digestion with a second and identical aliquot of proteases. To remove sodium deoxycholate and desalt peptides, two sample volumes of isopropanol containing 1% TFA were added to the digests, and the samples were desalted on a strong cation exchange (SCX) plate (Oasis MCX; Waters Corp., Milford, MA, USA) by centrifugation. After washing with isopropanol/1%TFA, peptides were eluted in 250 µl of 80% MeCN, 19% water, and 1% (v/v) ammonia; (2) Liquid chromatography-tandem mass spectrometry: Eluates after SCX desalting were frozen, dried, and resuspended in variable volumes of 0.05% trifluoroacetic acid and 2% acetonitrile to equilibrate concentrations. Approximately 1 µg of each sample was injected into the column for nanoLC-MS analysis. (3) MS and MS data analysis: Dependent LC-MS/MS analysis of the TMT sample was performed on a Fusion Tribrid Orbitrap mass spectrometer (Thermo Fisher Scientific) interfaced through a nano-electrospray ion source to an Ultimate 3000 RSLCnano HPLC system (Dionex). Peptides were separated on a reversed-phase custom-packed 40 cm C18 column (75 µm ID, 100 Å, Reprosil Pur 1.9 µm particles, Dr. Maisch, Germany) with a 4-76% acetonitrile gradient in 0.1% formic acid (total time 140 min). Full MS survey scans were performed at 120'000 resolution. A data-dependent acquisition method controlled by the Xcalibur 4.2 software (Thermo Fisher Scientific) was used to optimize the number of precursors selected ("top speed") of charge 2+ to 5+ while maintaining a fixed scan cycle of 1.5s. The precursor isolation window used was 0.7 Th. Full survey scans were performed at a 120'000 resolution, and a top speed precursor selection strategy was applied to maximize acquisition of peptide tandem MS spectra with a maximum cycle time of 0.6s. HCD fragmentation mode was used at a normalized collision energy of 32% with a precursor isolation window of 1.6 m/z, and the MS/MS spectra were acquired in the ion trap. The peptides selected for MS/MS were excluded from further fragmentation for 60s. Tandem MS data were processed using MaxQuant software (version 1.6.3.4) 81 incorporating the Andromeda search engine 82. UniProt reference proteome (RefProt) databases for Homo sapiens and mice were used, supplemented with sequences of common contaminants. Trypsin (cleavage at K, R) was used as the enzyme definition, allowing for two missed

cleavages. The carbamidomethylation of cysteine was specified as a fixed modification. N-terminal acetylation of proteins and oxidation of methionine are specified as variable modifications.

Mitochondrial monitoring

An inverted microscope (Nikon Eclipse Ti-2) equipped with an Okolab cage incubator (H201-T-UNIT), 40x magnification, the Perfect Focus System (PFS), was used to capture 16-bit mosaic (5 × 5) images before and immediately after treatment as a baseline (BL), and then every 2 h for 3 days. Total MitoTimer [1] was segmented using a homogeneous area detection algorithm applied to combined red and green channels. Large objects that corresponded to the peri-somatic mitochondrial network were removed from the analysis considering only well-defined and distal mitochondria. Individual mitochondrial features (intensity of green, intensity of red, red/green ratio, elongation, area, length, skeleton branches, junctions, circularity, diameter, surface, shape factor, and mitochondria number) were measured and averaged in each frame. The shape factor is calculated from the area, and the convex hull perimeter ranges from 0 to 1, where 1 indicates a perfect circle. Finally, after log transformation of the averaged values, the change in the score relative to the first frame (baseline) was calculated. Psd95 signal was segmented using thresholding based on the background detection. VGLUT1 was segmented by spot morphological detection (typical diameter). To put out of consideration vglut1 volumes locating within Psd95 volumes, which might correspond to protein being transported rather than organized at an active zone, we generated a shell around psd95 from its subtraction to its own morphological dilatation (0.5um). The size was determined from the reported distance in literature (~0.1um), the limitations in our equipment's resolution, and the visual assessment of closely interacting punctas. For the analysis of mitochondrial ATP levels in neurons with neuronal Go-ATeam2 [2] expression, single mitochondrial units were semi manually segmented within 12 axons per replicate to conduct ratio measurements. The lentiviral was produced as described in the main methods, but expressing mito-GO-ATeam2 biosensor[3] under PGK promoter.

Supplemental References

1. Richetin K, Steullet P, Pachoud M, Perbet R, Parietti E, Maheswaran M, et al. Tau accumulation in astrocytes of the dentate gyrus induces neuronal dysfunction and memory deficits in Alzheimer's disease. Nat Neurosci. 2020;23:1567–79.

2. Nakano M, Imamura H, Nagai T, Noji H. Ca(2+) regulation of mitochondrial ATP synthesis visualized at the single cell level. ACS Chem Biol. 2011;6:709–15.

3. Spurlock B, Gupta P, Basu MK, Mukherjee A, Hjelmeland AB, Darley-Usmar V, et al. New quantitative approach reveals heterogeneity in mitochondrial structure–function relations in tumor-initiating cells. J Cell Sci. 2019;132.

4. Vevea JD, Wolken DMA, Swayne TC, White AB, Pon LA. Ratiometric biosensors that measure mitochondrial redox state and ATP in living yeast cells. J Vis Exp [Internet]. 2013; Available from: https://pubmed.ncbi.nlm.nih.gov/23912244/

Supplementary Figures and tables

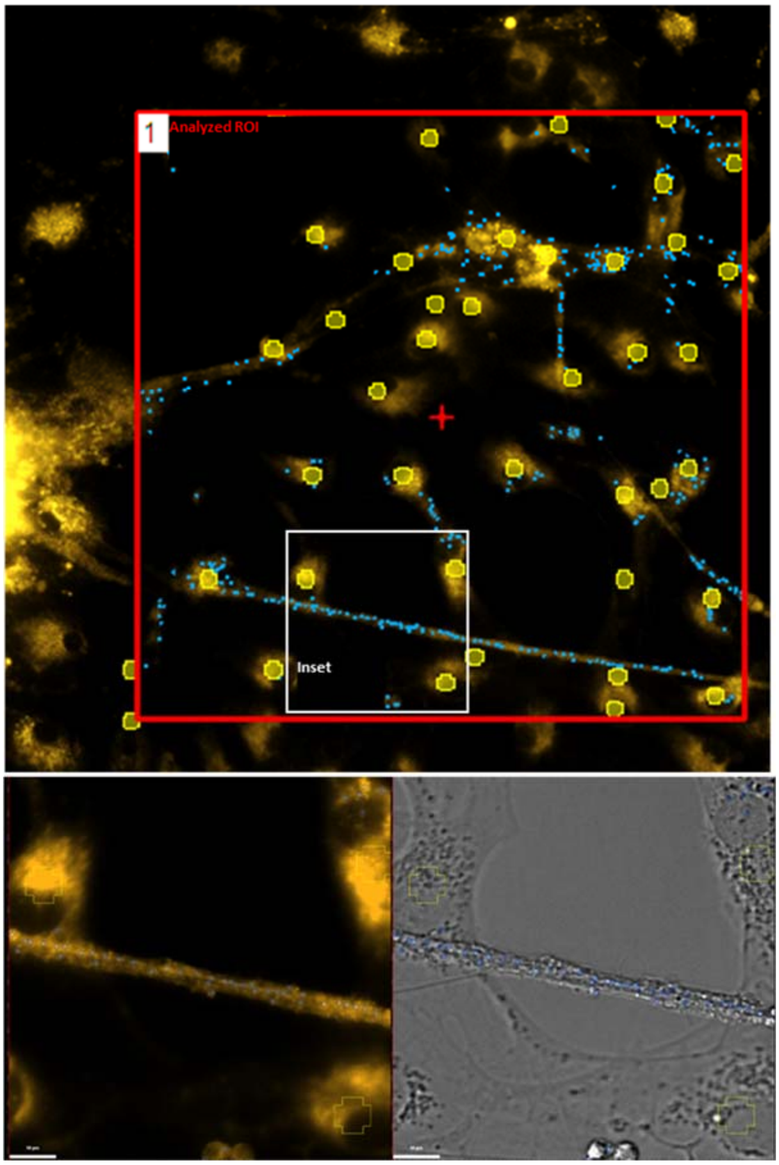

Figure S1. Analysis of synaptic vesicles density and cell body exclusion. Fluorescence microscopy image. An example of an analyzed ROI and its placement relative to neurospheroids (left side) is shown with a red square. Yellow circles represent detected cells bodies, surrounding areas of which were excluded in the Synaptic vesicles detection (blue dots). The white square shows the areas displayed in the bottom part with fluorescence and brightfield images.

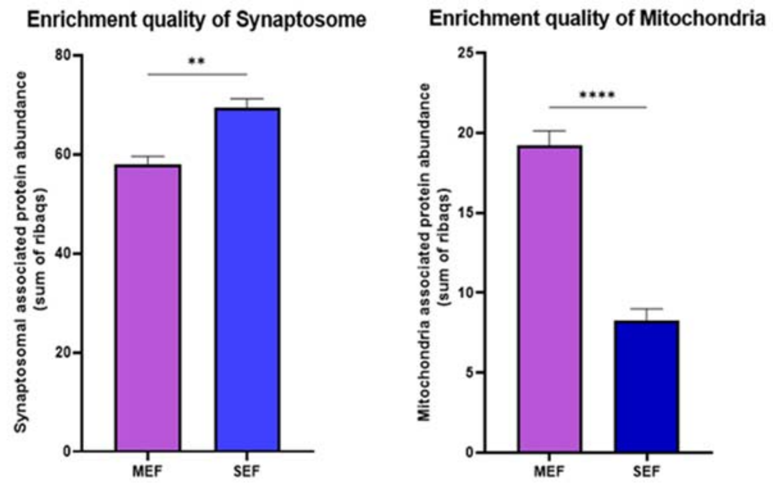

Figure S2. Enrichment of synaptosomal or mitochondrial proteins in SEF and MEF. Barplots of the total rIBAQs for synaptosomal (left) or mitochondrial (right) proteins in each fraction (MEF: purple, SEF: blue). Mean and SEM, ***P*<0.01, *****P*<0.0001, validating correct enrichment.

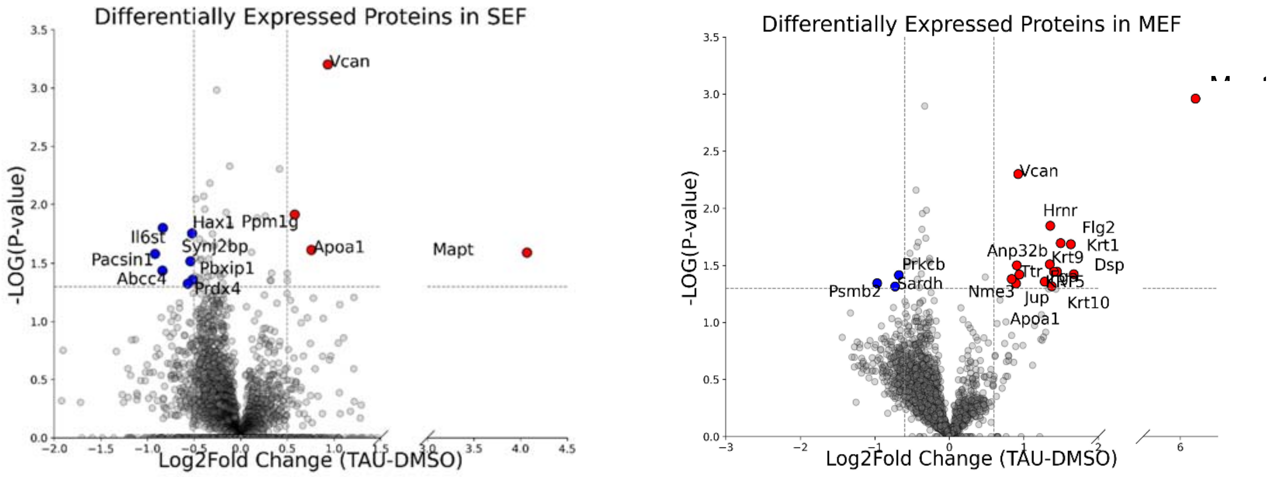

Figure S3. Volcano plots for SEF and MEF fractions. Differentially expressed proteins are represented with a volcano plot, with the most significantly and strongly regulated proteins highlighted. Proteins with large fold changes (>1.5) and significant *P*-values (<0.05) (e.g., MAPT, Apoa1m, Vcan) are labeled in red (enriched) or blue (depleted) depending on their regulation.

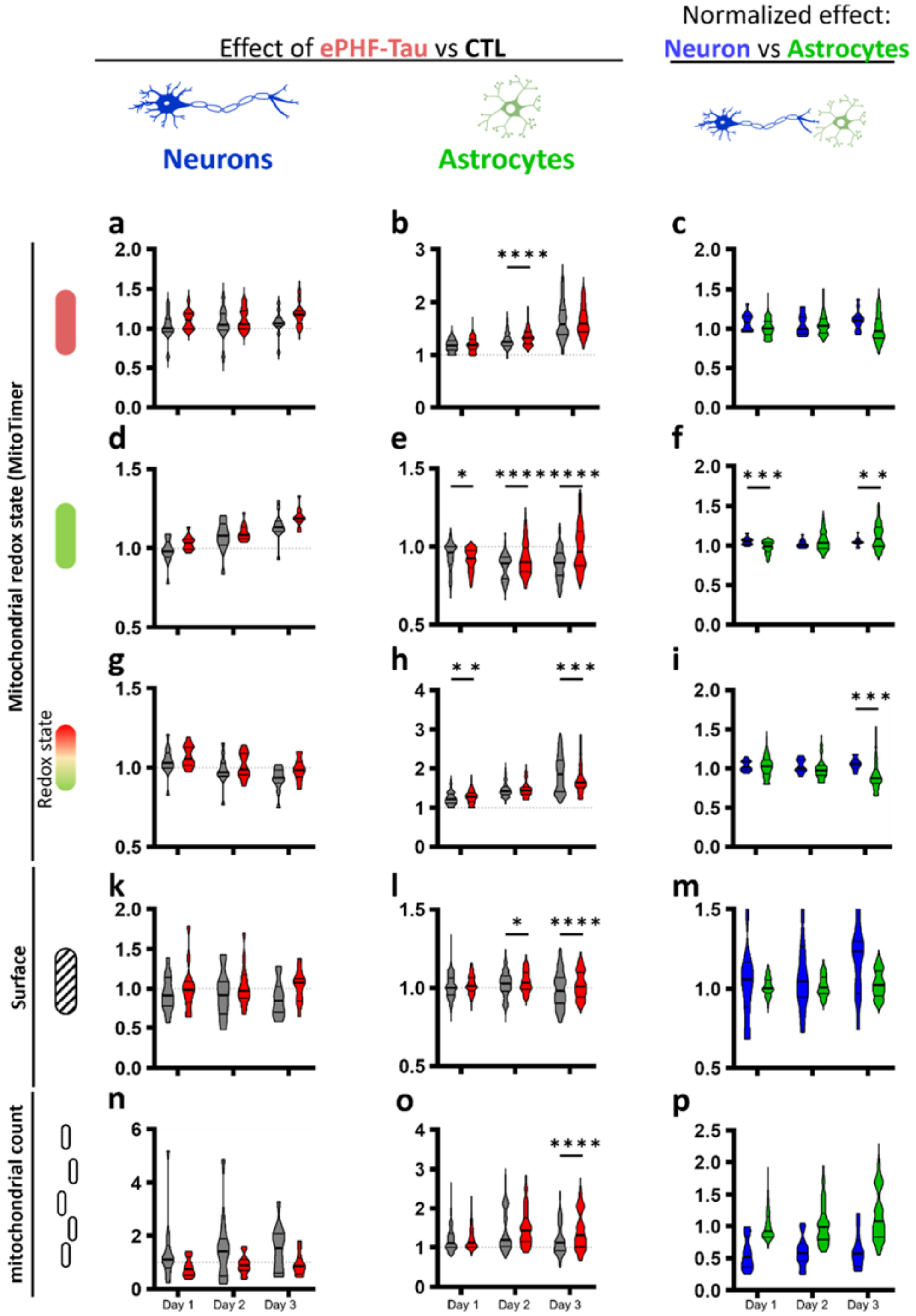
Effect of ePHF-Tau vs CTL
Normalized effect:
Neuron vs Astrocytes
Neurons
Astrocytes
a
b
c
d
e
f
g
h
i
k
l
m
n
o
p
Mitochondrial redox state (MitoTimer)
Redox state
Surface
mitochondrial count
Day 1
Day 2
Day 3

Figure S4. Mitochondrial feature quantifications pooled by days. **a-p** Comparative analysis of mitochondrial features aggregated by day, including turnover, redox state, surface area, and count, between neurons and astrocytes, shown as violin plots. Each row represents a specific feature: fluorescence intensity at 555 nm (1st row, **a**, **b**, **c**), 488 nm (2nd row, **d**, **e**, **f**), redox state (3rd row, **g**, **h**, **i**), mitochondrial surface area (4th row, **k**, **l**, **m**), and mitochondrial count (5th row, **n**, **o**, **p**). The left columns (**a, d, g, k, n**) show the effects of ePHF-Tau treatment (red violins) versus control (black violins) in neurons; the middle columns (**b, e, h, l, o**) depict the same comparison in astrocytes; and the right columns (**c, f, i, m, p**) compare neurons (blue) with astrocytes (green) relative to respective controls. Gray bars in each graph represent -log(*P*-value) for comparisons at each time point on the right y-axis. Asterisks above the graphs indicate *P*-values for comparisons over entire days (**P*<0.05, ***P*<0.01, ****P*<0.001, *****P*<0.0001).

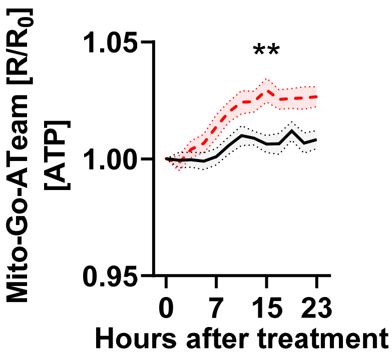

Figure S5. ATP monitoring in neuron after ePHF-Tau. Lineplot of mitochondrial ATP levels (Mito-Go-ATeam2 biosensor[4], R/R_0) in neurons with ePHF-tau treatment (red) and control (black) (mean value and SEM,***P* <0.01).

Table S1 Significantly enriched and depleted proteins in SEF

Protein	-LOG(*P*-value)	log2 Fold Change Tau-CTL
**Mapt**	1.591	4.069
**Vcan**	3.202	0.934
**Apoa1**	1.614	0.758
**Ppm1g**	1.913	0.579
**Pbxip1**	1.358	-0.514
**Hax1**	1.750	-0.521
**Synj2bp**	1.516	-0.539
**Prdx4**	1.324	-0.567
**Il6st**	1.797	-0.835
**Abcc4**	1.436	-0.839
**Pacsin1**	1.579	-0.919

Proteins modified in the Synatosome enriched fraction with transformed *P*-values and fold-changes

Table S2 Significantly enriched and depleted proteins in MEF

Protein	*P*-value	Log fold-change	Brain cell specificity	Functional annotations
**Mapt**	0.001	6.190	Neuron	Microtubule cytoskeleton organization and Neuron migration
**Dsp**	0.036	1.656	Neuron	Regulation of actin filament-based process and Macromolecule localization
**Flg2**	0.021	1.617	Unspecific	Structural molecule activity and Calcium ion binding
**Fga**	0.037	1.389	Unspecific	Regulation of vasoconstriction and complement and coagulation cascades
**Hrnr**	0.014	1.341	Astrocyte	Calcium ion binding activity and Transition metal ion binding activity
**Jup**	0.042	1.266	Unspecific	VEGFA-VEGFR2 Pathway and Neutrophil degranulation
**Ttr**	0.035	0.927	Microglia	Purine-containing compound metabolic process and Thyroid hormone metabolic process
**Vcan**	0.006	0.924	Astrocyte	Glycosaminoglycan metabolism and Heparan sulfate/heparin (HS-GAG) metabolism
**Anp32b**	0.028	0.906	Oligodendrocyte	Vasculature development and Ventricular system development
**Apoa1**	0.040	0.896	Endothelial	NADPHX epimerase activity and Racemase and epimerase activity
**Nme3**	0.037	0.835	Unspecific	Purine and Pyrimidine nucleotide biosynthetic process and Mitochondrion organization
**Prkcb**	0.033	-0.678	Neuron	Glutamate binding and activation of AMPA receptors and synaptic plasticity
**Sardh**	0.045	-0.728	Astrocyte	Metabolism of amino acids and derivatives and Oxidoreductase activity
**Psmb2**	0.049	-0.979	Astrocyte	Cellular response to hypoxia and Cross-presentation of soluble exogenous antigens (endosomes)

Proteins modified in the mitochondrial enriched fraction with indication of *P*-value, log fold-change, brain cell specificity and functional annotations.
